# “Watching Eyes” Triggers Third-Party Punishment: The Role of Emotion Within the Eyes

**DOI:** 10.3389/fpsyg.2021.681664

**Published:** 2021-07-15

**Authors:** Mingping Li, Chenyu Shangguan, Huqing Shi, Jiamei Lu

**Affiliations:** ^1^Department of Psychology, Shanghai Normal University, Shanghai, China; ^2^College of Education Science and Technology, Nanjing University of Posts and Telecommunictions, Nanjing, China

**Keywords:** watching eyes, third-party punishment, emotion, reputation, eye cues

## Abstract

Third-party punishment refers to a behavioral phenomenon whereby people punish wrongdoers even if their sanction incurs personal costs but yields no direct benefits. Given the eye cues demonstrated ability to convey signals of being observed, its effect on third-party punishment, driven by virtue of its effects on others' perceptions, was investigated. In addition, emotional message featured in the eye region is crucial in social interaction, whether the emotion within the eyes serves this effect with varying degrees of influence has rarely considered. The present study aimed at exploring (a) the watching eyes effect on the third-party punishment and (b) whether this effect varies from negative eyes to positive eyes. By two experiments using a modified Third-Party Dictator Game, we displayed either eye images or control images above the question on whether to punish the dictators or not. There was no emotional diversity of eye cues in Experiment 1, and most participants tended to punish for unfair offer. However, the appearance of eye images increased the punishment relative to control images. In Experiment 2, the eye cues were subdivided into positive and negative. The effect of watching eyes on the third-party punishment was significantly stronger when the eyes were negative than positive. Results revealed that eye cues play a role in promoting the third-party punishment and offer a potential insight into the mixed findings, such that the emotion within the eyes, especially the negative expression in the eyes, may influence the watching eyes effect.

## Introduction

Third-party punishment (TPP), a behavior phenomenon that occurs in the situation of norm violation when the violator is punished by an individual whose interests have not been harmed (Fehr and Gächter, 2002; Fehr and Fischbacher, 2004a; Rodrigues et al., 2020), appears cross-culturally (Henrich et al., 2006), and in different ages (McAuliffe et al., 2015; Yang et al., 2018). It's obvious that punishing wrongdoers entail significant costs, as the time, money, and energy are expended, and the punisher may also be backfired occasionally (Dreber et al., 2008; Nikiforakis, 2008; Balafoutas et al., 2014). Nevertheless, people paying a cost to inflict punishment is common.

Numerous studies examining the potential motivations underlying TPP have suggested that people penalize for more self-oriented reasons as well as social norms maintenance (Rodrigues et al., 2020). One well-known finding in recent decades is that the visibility of third parties' punitive actions may affect their punishment behavior (Kamei, 2018), implying such behavior is designed to benefit the individual because of its effects on others' perceptions. Models are driven by reputation effect, such as the costly signaling model, suggesting the cognitive mechanisms underlying TPP might have evolved because of their signaling benefits (Johnstone and Bshary, 2004; Kurzban et al., 2007), that is, individuals accept abandoning self-interests to inflict punishment in order to gain a positive appreciation with observers. As it's unlikely to bring direct benefits to the punisher, and TPP is usually beneficial to other group members, therefore, punitive actions are essentially equivalent to showing good qualities (Jordan and Rand, 2019; Chen and Yang, 2020), such as fairness and generosity (Nelissen, 2008), being trustworthy (Jordan et al., 2016), or willing to sacrifice for others (Jordan and Rand, 2017), and through the punitive action, TPP can, in the long run, help the punisher build a good reputation and improve the probability of getting help from others (Chen and Yang, 2020).

As a special clue, eyes can convey social information served as an implicit signal of being observed. Growing evidence suggests that people tend to adjust their behavior when a picture of eyes or stylized eye images is presented with eyes looking straight ahead (hereafter: eye cues, Bateson et al., 2006; Rigdon et al., 2009; Nettle et al., 2013). This effect, called the “watching eyes effect,” suggests that just feeling watched may be able to make people modify their actions unconsciously (Keith et al., 2019). Although the eye cues have been shown to stimulate a great variety of cognitive processes and behaviors (Conty et al., 2016), whether the eye cues may also play a role in altering TPP was unknown. One interpretation on what triggers the watching eyes effect comes from “norm psychology,” which demonstrates that individuals are sensitive to specific behavioral norms and tend to sanction deviation from these norms (Kallgren et al., 2000; Chudek and Henrich, 2011; Bateson et al., 2013). The interpretation is that pro-social acts are performed since eye cues enhanced the adherence to norms via the awareness of others (Oda et al., 2015). Fairness is a social norm, and people tend to follow fairness norms and are willing to pay a cost to sanction fairness violations (Fehr and Fischbacher, 2004a; Chen et al., 2020). Given this logic, it's reasonable that eye cues, offering a rich signal value, might well-lead people to inflict punishment and help them build a positive reputation. Indeed, evidence demonstrates that even without having eye images, people impose costly TPP when their punishment acts are made known by others (e.g., Kurzban et al., 2007; Kamei, 2018), whose intention may be driven by similar physiological effects to watching eyes effects. Accordingly, we assumed that third parties take more punitive action against those who violated fairness norms when being “watched” by eye images.

The watching eyes effect was initially demonstrated setting stylized eye spots on a computer screen (Haley and Fessler, 2005), but other studies operating such effects by posting eye posters on the wall (Bateson et al., 2006), or displaying robot eye graphics (Burnham and Hare, 2007), or subtle artificial eye images (Baillon et al., 2013), or even three dots similar to human faces (Rigdon et al., 2009; Xin et al., 2016) on the computer screen. However, different settings of eye cues may be responsible for inconsistent watching eyes effect (e.g., Raihani and Bshary, 2012; Bush et al., 2016; Northover et al., 2016); for example, an image of watching eyes did not decrease dishonest behavior (Cai et al., 2015) and did not increase the charitable giving in field research (Ekström, 2011). The main interpretation on why eye cues can increase pro-sociality is that one's behavior observed by others entails social consequences (Oda et al., 2011; Powell et al., 2012). Moreover, researchers stated that human eyes are unique to enhance the signal of being watched, which makes human eyes an important tool for communication (Kobayashi and Kohshima, 2001). Human eyes as a critical region to convey rich emotional and social information can express and recognize the complex mental states, generally called “language of the eyes” in the literature (e.g., Baron-Cohen et al., 1997). To date, empirical studies of how individuals modify their actions when feeling watched have neglected the emotion expressed in their eyes. Emotion-associated eyes, which are frequently used in social interactions, refer to those containing emotional messages and expressing mental states (Wagenbreth et al., 2014). Given that emotion-associated eyes can induce automatic implicit emotional processing (Fox and Damjanovic, 2006; Wagenbreth et al., 2014), it is reasonable to speculate that the watching eyes effect occurs, not only because the appearance of eye cues makes us feel watched, but also because another aspect of eye cues is playing a part. Therefore, in this study, we used ecological valid human eye pictures and further examined whether the emotion within eyes serves watching eyes effect with varying degrees of influence.

Previous studies demonstrated that the punitive action to norm violations is under the influence of the perceived emotional facial expressions of others (Mussel et al., 2018). Given the fact that eyes can make people aware of the existence of others and accordingly, it is unsurprising that different emotion-associated eyes lead a varying influence on watching eyes effect. In agreement with the perspective from reputation-based partner-choice models that pro-social behavior is conducted with the expectation of being chosen for reciprocal interactions in the future (Roberts, 1998; Barclay, 2004; Sylwester and Roberts, 2010), partner-choice makes pro-social behaviors worth performing. Eyes have evolved to be valid cues, which increase the likelihood of future partner-choice. Negative eyes (e.g., eyes expressing anger), which may convey negative social meaning such as blaming or intimidating, usually are understood as a message of non-approval and confrontation in terms of their actions (Hess et al., 2000; Rozin and Royzman, 2001; Horstmann, 2003; Fox and Damjanovic, 2006). Therefore, it is possible that negative eyes are capable of motivating individuals to perform pro-social behaviors to avoid such blame and increase the likelihood of future partner-choice. In this regard, we assumed that the presence of eyes with negative expression, implying the possibility of being blamed or criticized, would increase punishment on violators than positive-associated eyes.

To summarize, theoretical models and experimental evidence in this area support the view that people are concerned about impressions on others, and this may potentially influence third parties' punishment behaviors. The question remained whether the watching eyes effect, always been explicated as a response that reputation is at stake (Oda et al., 2011), was also occurred to TPP. Furthermore, it is also unclear whether the emotion expressed in the eyes causes a varying watching eyes effect. In this research, we conducted two experiments to investigate the impact of eye cues on TPP and the role of emotion within eyes in watching the eyes effect. We adapted the Third-Party Dictator Game (TP-DG) task in the experiment, which is typically used by Fehr and Fischbacher (2004b), wherein the participants made a decision to either punish or non-punish the dictator.

Therefore, the primary aim of the current study was to investigate the influence of eye cues on TPP. Specifically, we expected that the presence of eyes would increase the punitive actions of those who proposed unfair offers (Hypothesis 1). The secondary aim was to examine whether eyes within specific emotions, such as happiness or anger, will have a different impact on the effects of watching eyes on TPP. Consistent with Mussel et al. (2013), which demonstrated that positive and negative facial expressions of others would have different influences on decision making, we expected that eyes within the negative emotions would result in more punitive actions than positive emotions (Hypothesis 2).

## Experiment 1

### Method

#### Participants

A total of 56 college students were recruited using posters on campus and online forum. When recruiting participants, they were told that the initial remuneration was CNY 15, but the final amount was related to their performance in the money game, the more coins they saved, the more they got paid (in fact, the final payoff each person received after the experiment was CNY 20, about USD 3.12, which is not related to the coins they saved. See 2.1.3 Design and Procedure part for details). As there is a data storage error of one person, 55 participants' data (40 females, 15 males) were used in our final analysis. They were aged between 20 and 26, with an average age of 23.28 (*SD* = 1.37 years), and all of them were right-handed, with normal or corrected vision. Each participant read and signed the informed consent before the experiment. Ethical approval for the experiment was obtained from the Ethical Committee of the School of Psychology at Shanghai Normal University.

#### Materials

Images of eyes, as stimulus materials, were extracted from photographs of faces picked from the Chinese Facial Affective Picture System (CFAPS, Gong et al., 2011). For the present experiment, we chose six different persons out of CFAPS (three male, three female) with neutral facial expressions. The pictures were then adjusted and cut so that the eye regions were visible, which finally was presented in a size of 220 × 80 mm. Then we invited 29 college students who did not participate the formal experiment rated the arousal for each of the pictures on a scale of “1” (low) to “7” (high arousing) and valence on a scale of “1” (extremely unpleasant) to “7” (extremely pleasant). The results showed the arousal of pictures of eyes (3.31 ± 1.88) and clouds (3.08 ± 1.83) and the valence of eyes (3.58 ± 1.30) and clouds (3.94 ± 1.38). Paired *t*-test revealed that there was no difference between the eyes and clouds, whether arousal or valence (*ps* > 0.05).

#### Design and Procedure

The design was a two-factor within-subject design. One refers to the eye cues (picture with eyes or control picture), and the other refers to the degree of the unfairness of the dictator's decision (50:50–100:0). Our experiment adopted a modified TP-DG task with a total of 100 coins. Proposer A decides how to place 100 coins between A and recipient B. No matter how A decides the allocation, B can only accept it. The subject C, as the third-party, has 50 coins at the beginning of each penalty game, and the first witnessed the distribution of 100 coins between two players. If the subject feels that the distribution plan given by A is unreasonable, he/she could punish A; if so, C will pay 15 coins and A will lose triple coins as a result; if C agrees with the offer, he/she can keep all the coins, and the coins earned by A and B will be distributed according to A's proposal. In this experiment, A and B are virtual characters, and all allocation plans are presented randomly by a computer. Six types of splits in the experiment characterize equitable sharing (i.e., 50:50) to complete selfish (i.e., 100:0). Punishment in this game is costly because participants were informed that the coins they saved are linear related to the extra cash rewards (a maximum of CNY 10) in final payoff.

**Figure 1:**
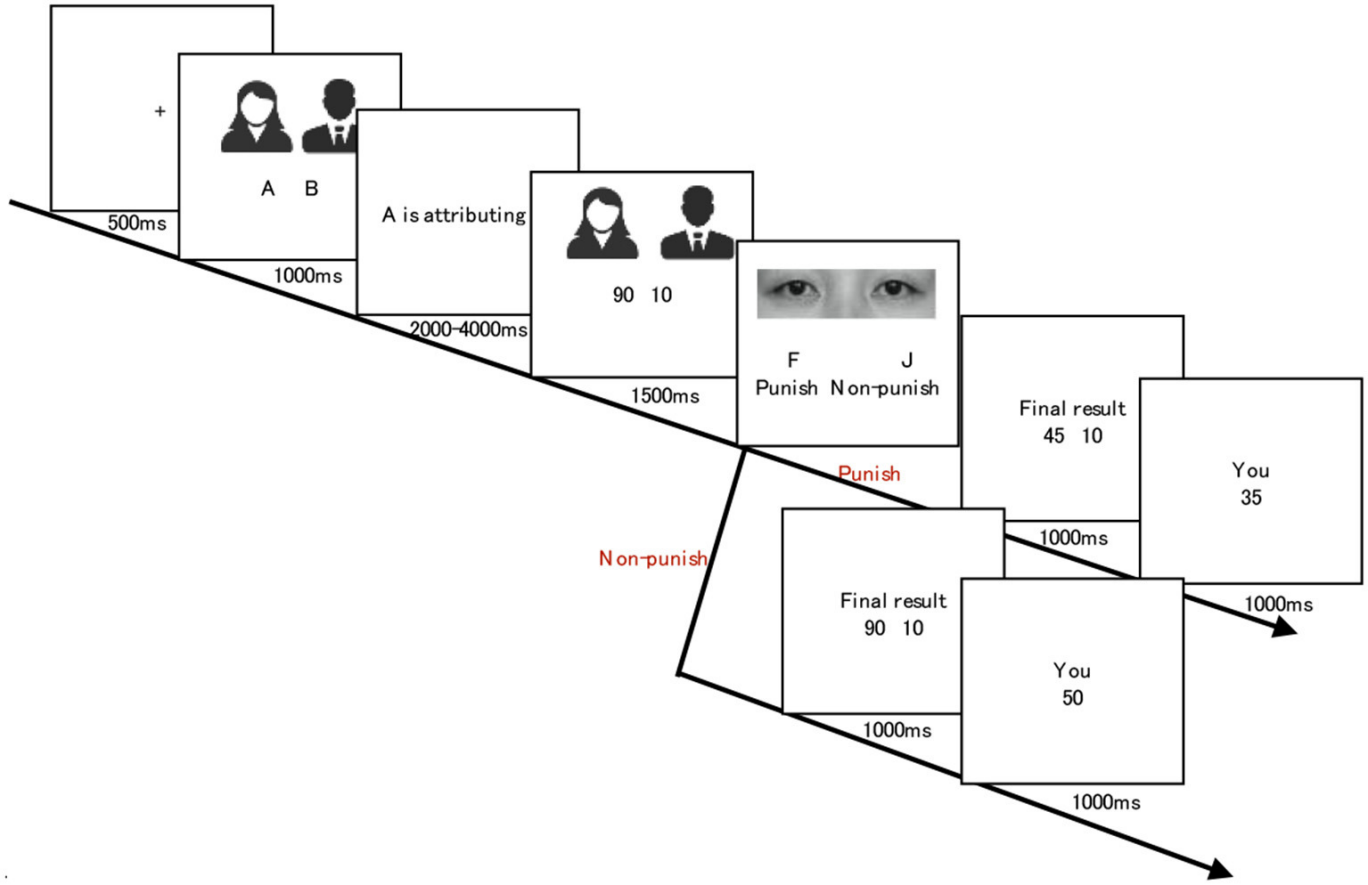
Example of one trial. The picture shows in eyes condition, results of punish or non-punish.

The experiment was carried out one by one. Upon arriving at the quiet laboratory, each participant was seated in an individual cubicle about 60 cm in front of the computer monitor. Figure 1 shows the structure of one experimental game (taking a 90:10 allocation plan as an example). In each round of the game, a fixation of 500 ms was first shown and then anonymous avatar A (dictator) and B (recipient) were presented for 1,000 ms. To balance the possible influence of gender, male and female photos will alternately be presented as dictators and recipients, and each pair of photos of A and B matches the split (50:50–100:0). After witnessing the initial allocation scheme of 100 coins between two players, a response stage of F/J underneath the watching-eyes picture was shown, at which the participants need to execute the decision they made according to the instruction to press keys (“F” for Punish and “J” for Non-punish) without time limitation. Finally, the numbers of coins obtained by A, B, and subjects are presented separately with a time of 1,000 ms. This is one trial of the task. The formal experiment was arranged in two blocks, and there were 36 trials in each block. Eye cues were equally probable (36 trials each), while cloud cues were also equally probable (36 trials each), and the entire experiment would take up to 15 min for the participants. After each block, the participants were required to answer the question “During the experiment, did you notice any eyes pictures?”, as a manipulation check for eye cues, on a 7-point scale: “1” = no attention at all, “7” = entirely noticed. After finishing the whole experiment, they had enough time to get rest.

### Results and Discussion

#### Manipulation Check

A one-sample *t*-test was used to test whether the manipulation is effective. The result showed that mean attention of eyes was higher (*M* ±*SD* = 4.82 ± 1.06) than the midpoint (4) of the 7-point Likert scale, *t*_(54)_ = 5.78, *p* < 0.001, which means that the eye cues were really noticed by participants.

#### Third-Party Punishment

**Figure 2:**
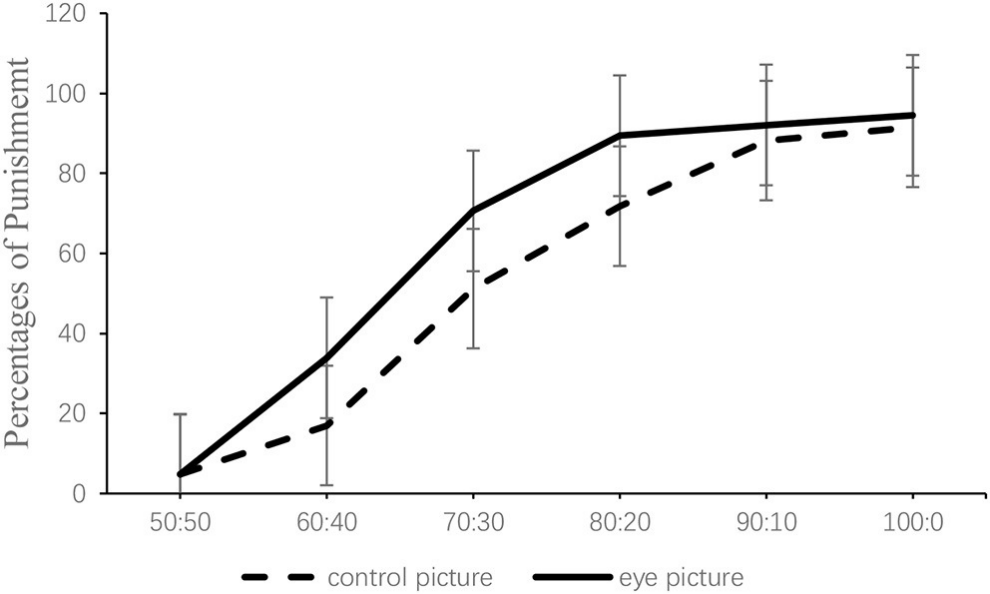
Percentages of punishment as a function of eye cues and the dictator's splits in Experiment 1. Error bars represent the 1-σ standard deviation intervals.

Across the sequential TP-DG tasks, each and every split from 50:50 to 100:0 occurred 660 times, half of which are eye pictures and the other half are control pictures. The percentages of punishment decisions followed a similar increase for both cues as decisions of the dictator became more selfish [$\chi_{\left(5\right)}^{2}$ = 202.09, *p* < 0.001, see Figure 2]. Our core hypotheses is that the punitive behavior in eyes pictures could be more than that in control pictures. In the punishment round, there are 48% of punishment decisions in the control pictures, while there are 63% of punishment decisions in the eye pictures. The picture of eyes strongly increased the proportions of punishment (Mann–Whitney, *z* = 2.46, *p* = 0.014).

Considering that the experiment is based on a within-subject design, we then analyzed parameters studying the role of eye pictures by carrying out binary logistic regressions with the decision (to vs. not to punish the dictator) as the dichotomous criterion variable, and eye cues, splits, and gender as categorical predictors. Results (see Table 1) showed a main effect of splits, Wald (5) = 572.74, *p* < 0.001. The main effect of interaction was significant, Wald (5) = 11.48, *p* < 0.05. Neither the gender effect nor the main effect of eye cues was significant (*ps* > 0.05).

**Table 1:** The logistic regressions analysis of punishments in Experiment 1.

		**OR (95%CI)**	***P*-value**
**Gender**
	Female	1.00	
	Male	0.88 (0.73–1.06)	=0.18
**Splits**
	50:50	1.00	
	60:40	4.01 (2.25–7.16)	<0.001
	70:30	20.63 (11.94–35.65)	<0.001
	80:20	50.13 (28.73–87.48)	<0.001
	90:10	146.86 (80.32–268.54)	<0.001
	100:0	212.32 (112.59–400.39)	<0.001
**Eye cues**
	Control picture	1.00	
	Eye picture	1.00 (0.49–2.04)	=1.00
**Eye cues** * **Splits**
	By 50:50	1.00	
	By 60:40	2.52 (1.13–5.60)	<0.05
	By 70:30	2.29 (1.05–4.99)	<0.05
	By 80:20	3.31 (1.45–7.57)	<0.01
	By 90:10	1.57 (0.65–3.78)	=0.32
	By 100:0	1.61 (0.63–4.11)	=0.32

The aim of the present experiment was to test whether the presentation of eye cues would elicit greater TPP than other cues. Consistent with prior research, with the increasing degree of inequality, third parties tended to punish allocators more, even they must pay a price (Fehr and Fischbacher, 2004a; Sun et al., 2015). However, eye cues appeared to influence TPP, in that participants in a modified TP-DG task inflict significantly more punishment when “watched” by eyes picture. In particular, individuals exposed to the pictures of eyes were more likely to punish the dictators under the splits at 60:40, 70:30, 80:20, with 2.52, 2.29, 3.31 times penalty rate, respectively, than participants exposed to the control pictures (supported Hypothesis 1).

These results replicated the third-party punishment and extended the boundary of the watching eyes effect on pro-social behavior (Haley and Fessler, 2005; Powell et al., 2012) to the third-party punishment. In the task, participants were informed that they can keep the extra money they gain during the game, and they can also spend their own coins to alter the unfair allocation to reach the fair. According to the mainstream economic model of the self-regarding or behavioral economic model of other-regarding preference, there would be no difference of punishment either with the picture of eyes or with the control picture (Bolton, 1991; Ledyard and Palfrey, 1994; Fehr and Gächter, 2002; Fehr and Fischbacher, 2004a; Kim et al., 2013). Surprisingly, watching eyes did promote third party's irrational punishment, even though there is a conflict between a desire for fairness and self-interest. In this way, our results could be taken as evidence that eye cues promote the disapproval of unfair, selfish acts, consistent with prior work showing that people impose costly TPP when their punishment acts are made known by others (e.g., Kurzban et al., 2007; Kamei, 2018). The reason why eye cues are effective in increasing TPP can be interpreted with the main motivation of third-party behaviors, maintaining the social norms. Based on the punishment under the unfair offer, it could be inferred that the eye cues promote the disapproval of unfair, selfish acts. It can be interpreted that unfair distribution is a kind of violation of the norm of fair sharing. The presence of eyes can capture attention naturally and increase adherence to norms (Oda et al., 2015). Therefore, eye cues appeared to intensify the punishment for norm violation. However, this effect only occurred when the dictator's decisions are less selfish. It can be interpreted that, in the cases of complete fairness (50:50), there is no need to make TPP, and in the case of extreme unfairness (100:0, 90:10), the behavior of punishment has a ceiling effect. The present study extends our understanding of pro-social behavior by the focus on social cues in TPP.

In sum, the association between the presence of eye cues and TPP was significant, which examined that the eye cues played a role in TPP. In Experiment 1, we focused on whether there were eye cues or not, but we did not take the emotions within the eyes into account. After the eye cues were subdivided, how would the positive and negative pictures of eyes affect TPP? Previous research has shown there is comprehensive variance to different emotional expressions. On this basis, we wonder what kind of emotional expression within the eyes particularly makes an impact on third parties. Two meaningful expressions are considered, “happiness” for positive and “anger” for negative. Anger is identified as an adaptable emotional reaction in the situation of violations for moral standards, fairness, or equality, also called moral outrage, which is effective to motivate individuals to maintain social norms (Batson et al., 2007; Van Doorn et al., 2014; Gummerum et al., 2016; Rodrigues et al., 2020). Therefore, we assumed that eyes within the negative emotions would result in more punitive actions than positive emotion, and we tested this assumption in Experiment 2.

## Experiment 2

### Method

#### Participants

We initially recruited 40 college students, and one was excluded from the data analysis because he figured out the purpose of the experiment. Hence, 39 participants (28 females, mean age = 23.0 years, *SD* = 1.77 years) entered our analysis. All participants were Chinese native speakers, who reported no physical or mental illness. The study protocol was approved by the Ethical Committee of the School of Psychology at Shanghai Normal University. Participants were told before the experiment that they would get basic monetary compensation of CNY40 and had a chance of receiving extra cash rewards, which is linearly related to the coins they saved during the game. However, all of them got CNY 50 after the experiment, regardless of the coins they saved in the game. Each of the participants signed informed consent prior to the beginning of the experiment.

#### Materials

The materials for Experiment 2 were nearly identical to those used in Experiment 1. A critical difference involved the eye cues with the emotion expressed in the eyes. Emotional-associated eyes, as stimulus material, were selected from CFAPS and grouped into two affective stimulus categories: positive (happy, *M*_arousal_ = 5.03 ± 1.52, *M*_valence_ = 4.51 ± 1.93) and negative expression (angry: *M*_arousal_ = 5.84 ± 1.27, *M*_valence_ = 2.91 ± 2.28). Each affective category contained six pictures. Paired *t*-test revealed that there was a significant difference between the valence for each eye [*t*_(28)_ =3.05, *p* < 0.01], and not significant between arousal for each eye (*p* > 0.05). More precisely, we invited 27 post-graduates to identify each emotional expression of eyes pictures. Participants indicated on a 5-point Likert scale (ranging from 1 = extremely disagree to 5 = extremely agree) to what extent they agree with the identification for happy or angry expression. A one-sample *t*-test results showed that mean agreement on happy expression was higher (*M* ±*SD* = 3.94 ± 0.74) than the midpoint (3) of the 5-point Likert scale, *t*_(26)_ =6.54, *p* < 0.001; and angry expression (*M* ± *SD* = 4.28 ± 0.63), *t*_(26)_ = 10.50, *p* < 0.001. The result shows that the emotional-associated eyes we extracted from the facial picture can indeed convey a specific emotion.

#### Design and Procedure

The design was a two-factor within-subject design. One refers to the eyes with emotions (positive, negative), and the other refers to the degree of the unfairness of the dictator's decision (50:50–100:0). The third-party punishment game followed the same format as before, with the exception that emotional cues interposed between the allocation stage and the response stage. The formal experiment was arranged in five blocks, and there were 60 trials in each block. Negative cues were equally probable (150 trial each), while positive cues were also equally probable (150 trial each). After each block, they had enough time to relax.

### Results and Discussion

Data from the TP-DG task showed that the third-party punishment increased as the offer becomes unfair χ${}_{\left(5\right)}^{2}$ = 132.98, *p* < 0.001, and that TPP of negative condition (45%) was significantly more than that of positive condition (33%), Mann–Whitney, *z* = −2.22, *p* = 0.027. To test for the main effects of emotion within the eyes and unfairness of dictator's decision, we performed a binary logistic regression with the decision (to vs. not to punish the dictator) as the dichotomous criterion variable, and emotion, splits, and gender as categorical predictors. Results (see Table 2) revealed a main effect of the splits, Wald (5) = 545.72, *p* < 0.001. There was a main effect of emotional condition, Wald (1) = 25.86, *p* < 0.001, and there was a main effect of gender, Wald (1) = 10.17, *p* < 0.01. Results also revealed a significant gender × splits interaction effect, Wald (5) = 53.05, *p* < 0.001, as well as gender × emotions interaction effect, Wald (1) = 12.91, *p* < 0.001. There were no significant interaction effects of the emotion and splits (*p* = 0.980).

**Table 2:** The logistic regressions analysis of punishments in Experiment 2.

		**OR (95%CI)**	***P*-value**
**Gender**
	Male	1.00	
	Female	1.59 (1.20–2.13)	<0.01
**Splits**
	50:50	1.00	
	60:40	5.56 (3.43–9.32)	<0.001
	70:30	42.05 (25.78–68.60)	<0.001
	80:20	181.09 (105.39–311.16)	<0.001
	90:10	117.77 (65.94–210.33)	<0.001
	100:0	64.60 (35.83–117.02)	<0.001
**Emotion**
	Positive	1.00	
	Negative	2.55 (1.77–3.66)	<0.001
**Emotion** * **Gender**
	By male	1.00	
	By female	0.70 (0.58–0.85)	<0.001
**Gender** * **Splits**
	By 50:50	1.00	
	By 60:40	0.63 (0.45–0.88)	<0.01
	By 70:30	0.46 (0.33–0.63)	<0.001
	By 80:20	0.36 (0.25–0.51)	<0.001
	By 90:10	0.65 (0.43–0.96)	<0.05
	By 100:0	1.07 (0.70–1.62)	=0.77

Experiment 2 aimed to test whether the emotion within the eyes affected the watching eyes effect on TPP. First, a linear trend again showed that TPP would rise as splits became more selfish, and males would engage in greater third-party punishment than females except in the extremely selfish case. Second, we found the watching eyes effect was significantly greater when the eye cues were negative. Individuals exposed to negative eyes are 2.55 times more likely to punish than those exposed to positive eyes, and this negative effect was larger for males. Our novel hypothesis that the watching eyes effect on TPP was affected by the emotion within eyes was confirmed to some degree.

These results provide a first evidence that emotions expressed in the eyes influenced the watching eyes effect on TPP with varying degrees. Moreover, our results tentatively suggest that the eyes that contain emotional messages are particularly effective for eliciting moralistic punishment. The emotion-associated eyes in our experiment allowed us to examine whether the punitive action is influenced by the socioemotional cues. Angry eyes signal that others are angry about unfair distributions and lead to more punishment (Hess et al., 2000; Horstmann, 2003). One interpretation has been indicated based on the motivation of individuals to punish the norm violators (Van Doorn et al., 2014) that negative feelings of third parties, experienced as reactions to unfair sharing, motivates them to rebuild equality through punishment (Nelissen and Zeelenberg, 2009). Following this argumentation, emotion-associated eyes appeared to have the potential to induce a similar feeling to subjects. Thus, negative eyes would make participants increase TPP irrespective of the self-expense. As regards the finding of gender difference that females were less likely to punish in negative eyes than males, it might be speculated that males exposed to anger were more likely to trigger an angry or aggressive reaction. As such, we provide evidence that emotion expressed in the eyes leads to a varying watching eyes effect. The results extend previous research which neglected some certain factors like emotion. Watching eyes effect, as a peculiar phenomenon whereby the appearance of eyes will change behavior, does not appear under some circumstances. As emotional expression featured in eye regions is critical in social interaction, it is necessary to take further studies considering the difference of these emotional messages.

## General Discussion

In this research, we conducted two experiments to investigate whether the appearance of eye cues affects third parties' punishment on the violator of the norm of fair sharing (Experiment 1), and we attempted to examine whether the emotion within eyes affects watching eyes effect (Experiment 2). As far as we know, no previous research has investigated whether the eye cues influence TPP and the emotion within the eyes serve watching eyes effect with varying degrees of influence.

The results reported here clearly showed that the eye cues increased the frequency of punishment, even it requires immediate costs. At first glance, such fact might seem unexpected, but it matches with related findings showing that eye cues can engender behaviors that, though seemed to be problematic for themselves, actually work to enhance pro-sociality (e.g., Burnham and Hare, 2007; Sénémeaud et al., 2017). Prior work studying the decision-making process in the dictator game suggested that such decisions are led by two-step process. The players generate an automatic, intuitive proposal immediately and then go through a more deliberative phase, in which they adjust the initial proposal based on motivation and cognitive resources, a process that is influenced by social context (Cornelissen et al., 2007; Cappelletti et al., 2011; Rand et al., 2012). Following the two-step process model, our finding suggests that the watching eyes effect on TPP occurs in the second phase with the cognitive conflict. Results in this regard showed that eye cues lead to a greater punishment than other cues, that is to say, punishment tended to be automatically taken when it was likely to be witnessed. A potential explanation centers on pro-social motivation based on reputation (Mifune et al., 2010; Raihani and Bshary, 2015). Eye cues offering a rich signal value can capture attention nationally and trigger self-referential process, which induces individuals to moderate their behavior and unconsciously heighten concern over how they were socially evaluated (Oda et al., 2011; Conty et al., 2016). As such, people are more likely to sanction norm violations in order to gain a positive reputation and, consequently, intensify their TPP actions accordingly.

Concerning the finding that the watching eyes effect was significantly greater when the eye cues is negative, it can be explained by reputation psychology to some extent. People are willing to sanction those who break social norms (Fehr and Fischbacher, 2004a), which was regarded as a major driving force for maintaining pro-sociality in human societies (Gintis et al., 2003). Thus, individuals who did not respond to selfish or unfair behavior in the situation of norm violations will be identified as a lack of sense of justice (Gardner, 2019). In this way, the negative eyes appeared signaled negative appraisal about disregarding unfair behavior intentionally. An interpretation is based upon reputation-based partner-choice theories, which indicated that pro-social acts are performed to increase the probability of being chosen by others for reciprocal behavior in the future (Roberts, 1998; Barclay, 2004; Sylwester and Roberts, 2010). Considering that members of a group would interact with each other, in this way a reputation is transmitted interpersonally. A negative appraisal of someone may lead him not to be chosen in future interactions. Thus, individuals have evolved to be psychologically sensitive to negative appraisals (Baumeister et al., 2001) and tend to avoid negative signals about themselves for others to gossip about, rather than to seek to provide positive signals to be talked about positively (Keith et al., 2019). These factors may explain why participants take more pro-social behavior like punishing norm violators while they were “watched” angrily. Our results are consistent with the studies showing that the watching eyes effect promoted concerns about reputation, and people were more motivated to avoid a bad reputation than gain a good reputation (Oda et al., 2015). Admittedly, those interpretations are not consistent with the findings of Rigdon et al. (2009) and Xin et al. (2016) that even a simple dot pattern as weak social cues increased the individuals' pro-sociality, or the explanations of Pillutla and Murnighan (1996) and Yamagishi et al. (2012) that anger might trigger an angry or aggressive reaction. However, our results may explain why some studies have successfully found a meaningful effect (e.g., Nettle et al., 2012; manipulating the eye cues with the words: “Cycle thieves, we are watching you” underneath the angry eyes, a useful intervention against bicycle theft) and why eye spots do not increase generosity or altruism (e.g., Vogt et al., 2015; Northover et al., 2016). Thus, additional studies are needed to further explore the effects of watching eyes as well as its emotion on people's decision making.

In addition, this study has several limitations that we need to note briefly. First, when we observe one person treating another person unfairly, there are at least two options to react to this norm violation, either punishing the offender or helping the victim. But in our experiments, third parties could only decide to punish or not, we didn't take the third-party help into consideration. Previous studies indicated that, compared to third-party punishers, third-party helpers were more likely to be rewarded (Li et al., 2018). Therefore, future research can explore the watching eyes effects on the third parties upon help. In addition, in the second experiment, we merely compared “happiness” and “anger” expressions to see which emotion has more influence on the watching eyes effect. Because it did not include a condition of neutral emotion within the eyes as a control, it should be more cautious about explaining the main effect in the present study and should not be overinterpreted. Moreover, since there are a variety of human emotional expressions in social contexts, future studies should investigate this effect on more emotional expression categories.

To conclude, the current study uses a modified TP-DG task to demonstrate the watching eyes effect on the third-party punishment, and such effect is significantly greater while the watching eyes contain negative emotions than positive ones. We revealed for the first time that even eye cues could trigger third parties to take a punitive decision. We extended the boundary of the watching eyes effect on pro-social behavior to the third-party punishment, and moreover, we offered a potential insight into the inconsistent findings across both laboratory and field experiments of watching eyes effect that the emotion within the eyes, especially the negative expression in the eyes, may influence the watching eyes effect.

## Data Availability Statement

The original contributions presented in the study are included in the article/Supplementary Material, further inquiries can be directed to the corresponding author/s.

## Ethics Statement

The studies involving human participants were reviewed and approved by School of Psychology at Shanghai Normal University. The patients/participants provided their written informed consent to participate in this study.

## Author Contributions

ML: contributions to conceptualization, acquisition, collection, analysis, interpretation, and drafting. CS: contributions to conceptualization, interpretation. HS: contributions to interpretation and revision of the work. JL: contributions to supervision and validation. All authors contributed to the article and approved the submitted version.

## Conflict of Interest

The authors declare that the research was conducted in the absence of any commercial or financial relationships that could be construed as a potential conflict of interest.
