# Supplementary figures and images for: “Watching Eyes” Triggers Third-Party Punishment: The Role of Emotion Within the Eyes
Source: Front Psychol. 2021 Jul 15;12:681664. doi: 10.3389/fpsyg.2021.681664 (PMC8320698; doi:10.3389/fpsyg.2021.681664)

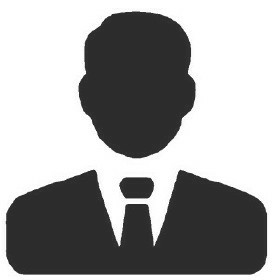

Supplement: Supplementary file 1 [file Data_Sheet_1.ZIP › 03 Materials and Data/Materials /m.jpg]

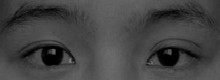

Supplement: Supplementary file 1 [file Data_Sheet_1.ZIP › 03 Materials and Data/Materials /e2.jpg]

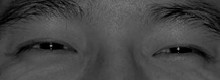

Supplement: Supplementary file 1 [file Data_Sheet_1.ZIP › 03 Materials and Data/Materials /p3.jpg]

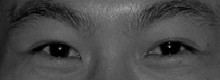

Supplement: Supplementary file 1 [file Data_Sheet_1.ZIP › 03 Materials and Data/Materials /p2.jpg]

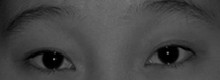

Supplement: Supplementary file 1 [file Data_Sheet_1.ZIP › 03 Materials and Data/Materials /e3.jpg]

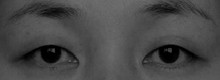

Supplement: Supplementary file 1 [file Data_Sheet_1.ZIP › 03 Materials and Data/Materials /e1.jpg]

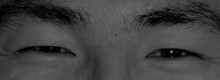

Supplement: Supplementary file 1 [file Data_Sheet_1.ZIP › 03 Materials and Data/Materials /p1.jpg]

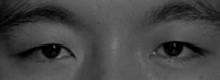

Supplement: Supplementary file 1 [file Data_Sheet_1.ZIP › 03 Materials and Data/Materials /e4.jpg]

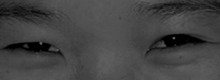

Supplement: Supplementary file 1 [file Data_Sheet_1.ZIP › 03 Materials and Data/Materials /p5.jpg]

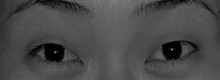

Supplement: Supplementary file 1 [file Data_Sheet_1.ZIP › 03 Materials and Data/Materials /p4.jpg]

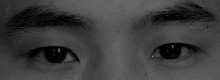

Supplement: Supplementary file 1 [file Data_Sheet_1.ZIP › 03 Materials and Data/Materials /e5.jpg]

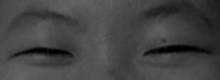

Supplement: Supplementary file 1 [file Data_Sheet_1.ZIP › 03 Materials and Data/Materials /p6.jpg]

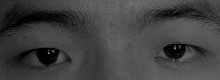

Supplement: Supplementary file 1 [file Data_Sheet_1.ZIP › 03 Materials and Data/Materials /e6.jpg]

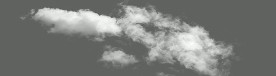

Supplement: Supplementary file 1 [file Data_Sheet_1.ZIP › 03 Materials and Data/Materials /c1.jpg]

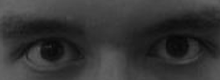

Supplement: Supplementary file 1 [file Data_Sheet_1.ZIP › 03 Materials and Data/Materials /n6.jpg]

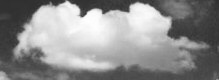

Supplement: Supplementary file 1 [file Data_Sheet_1.ZIP › 03 Materials and Data/Materials /c2.jpg]

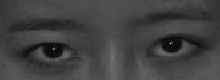

Supplement: Supplementary file 1 [file Data_Sheet_1.ZIP › 03 Materials and Data/Materials /n5.jpg]

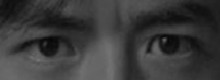

Supplement: Supplementary file 1 [file Data_Sheet_1.ZIP › 03 Materials and Data/Materials /n4.jpg]

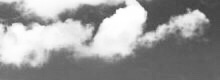

Supplement: Supplementary file 1 [file Data_Sheet_1.ZIP › 03 Materials and Data/Materials /c3.jpg]

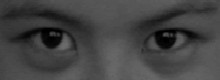

Supplement: Supplementary file 1 [file Data_Sheet_1.ZIP › 03 Materials and Data/Materials /n1.jpg]

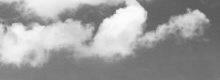

Supplement: Supplementary file 1 [file Data_Sheet_1.ZIP › 03 Materials and Data/Materials /c6.jpg]

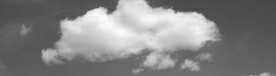

Supplement: Supplementary file 1 [file Data_Sheet_1.ZIP › 03 Materials and Data/Materials /c4.jpg]

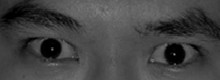

Supplement: Supplementary file 1 [file Data_Sheet_1.ZIP › 03 Materials and Data/Materials /n3.jpg]

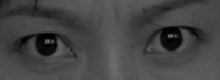

Supplement: Supplementary file 1 [file Data_Sheet_1.ZIP › 03 Materials and Data/Materials /n2.jpg]

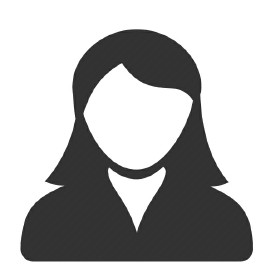

Supplement: Supplementary file 1 [file Data_Sheet_1.ZIP › 03 Materials and Data/Materials /f.jpg]

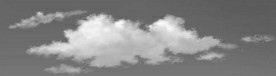

Supplement: Supplementary file 1 [file Data_Sheet_1.ZIP › 03 Materials and Data/Materials /c5.jpg]
